# Supplementary figures and images for: Impact of Lifestyle Modifications on Alterations in Lipid and Glycemic Profiles and Uric Acid Values in a Pediatric Population
Source: Nutrients. 2022 Feb 28;14(5):1034. doi: 10.3390/nu14051034 (PMC8912598; doi:10.3390/nu14051034)

## Supplementary Figure S1

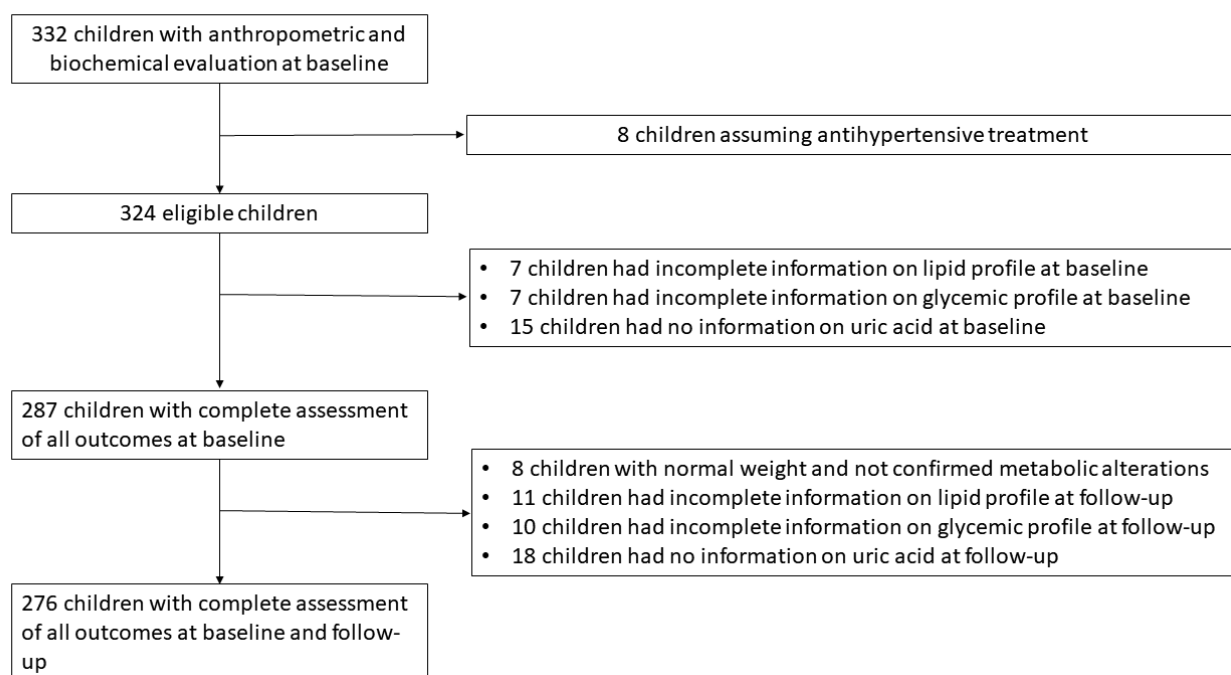

Please note: a few individuals had more than one data point missing.

Supplement: Supplementary file 1 [file nutrients-14-01034-s001.zip › nutrients-1598839-supplementary.pdf]
